# IDENTIFYING FACTORS ASSOCIATED WITH CAREGIVER NEGLECT IN OLDER PERSONS WITH DEMENTIA

**DOI:** 10.1093/geroni/igac059.3116

**Published:** 2022-12-20

**Authors:** E-Shien Chang, Sara Czaja, Tony Rosen, Jerad Moxley

**Affiliations:** Weill Cornell Medicine, New York, New York, United States; Weill Cornell Medicine, New York, New York, United States; Weill Cornell Medical College / NewYork-Presbyterian Hospital, Pelham, New York, United States; Weill Cornell Medicine, New York, New York, United States

## Abstract

Caregiver neglect is common among older persons with dementia and is associated with significant morbidity and mortality. Despite this, little empirical research exists examining factors that contribute or prevent neglect. Our goal was to identify caregiving factors that may be associated with caregiver neglect. Data were drawn from the baseline information of 240 caregivers enrolled in the Caring for the Caregiver Network study who provided care to a family member with dementia. Caregiver depression, burden, social support, perceived mutuality with care-recipients, positive aspects of caregiving, and self-perceived caregiving preparedness, were measured using validated scales. To maximize sensitivity in our measure of caregiver neglect, we operationalized caregiver neglect as consisting of: 1) caregivers’ failure to meet care-recipients’ needs, using 8-item instrumental activities of daily living (IADL) and 6-item ADL scales, and 2) caregivers not receiving additional formal services (e.g., visiting nurses; home care aides) to address care-recipients’ unmet needs. Selection of independent variables into multivariate regression models examining predictors of neglect was based on significance in bivariate analysis (p < .10). Caregiver neglect was found in 29.2% of caregivers. The caregiver being male was significantly associated with greater risk of neglect. In the final adjusted model, only caregiver preparedness was found to be a significant risk factor for neglect (β = -.16 SE=.10, p < .05). This analysis provides the first evidence of the association between caregiver preparedness and risk of caregiver neglect. Future research should examine possible psycho-social mechanism linking preparedness and neglect to inform neglect prevention and intervention programming.

